# Paralysis

**Published:** 1872-11

**Authors:** 


### PARALYSIS

Is a sudden loss of the power to move either the whole body, half of it, or some smaller portion; if of half the body, as of one side, it is called hemiplegia; if the lower part of the body, it is paraplegia. The common name is palsy; when the motions are present, it is shaking palsy.

There are two kinds of muscular motion, one voluntary, as in that of the hand or foot, the other involuntary, as the beating of the heart; the latter is beyond the control of the will; the other, in health, obeys its behests.

The involuntary motions of the interior of the system are not necessarily affected in ordinary paralysis, for the functions of digestion go on as before, and so does pulsation. It is often functional, when only the lower limbs are affected.

The power of motion comes from the brain and from the spinal cord, called by some the spinal marrow, which is an actual prolongation of the brain, extending to the lower end of the backbone; it really passes through the whole extent of the backbone, through a bony canal, or rather tubular way, so as to be more perfectly protected against injury.

When the brain is in health and power, it sends its forces through the nerves to every part of the system, accompanying that power with the will to exercise it.

The brain is fed, is nourished, by the nutriment contained in the blood, which is sent to it fresh and healthful at every beat of the heart; and is so dependent for its life on that, that if for one instant of time the heart ceased to beat, ceased to send its supply of sustenance-giving blood, that instant would the man fall to the earth, as dead as in a common fainting-fit.

Paralysis, then, is the result of the brain failing to give the power of motion to the voluntary muscles, that failure arising from want of nourishment.

Occasionally there is paralysis from some obstruction occurring between the brain and the muscles, as if a nerve is cut in two, like the break in a cable, when it ceases to bear messages; the messages are sent, but they go only as far as the break; or there may be some diseased growth along the line which absorbs, eats up the line at that part, or so presses against it as to interrupt healthful action. But it is the ordinary interruption of voluntary muscular motion which is here treated, the cause being want of nourishment in the brain, which may arise from various causes; among the most prominent are,—

First. A want of general good health. None of the blood is pure, healthful, nutritious; hence the brain wastes and gradually loses its power. Idiocy attends starvation.

Second. But the paralysis which usually goes by that name comes on from another cause; comes suddenly as the lightning's stroke; the machine ceases to work as instantaneously as a watch, when one of its wheels is broken, although it worked perfectly up to the very instant of the breakage. In common paralysis, there are sometimes indications of a coming attack, in a feeling of numbness in some of the limbs, and others to be spoken of shortly.

Third. Paralysis may be caused by excessive stimulus of the brain; it may be overworked by either thinking or drinking. Alcohol in any form goes directly to the brain — is first felt in the head. Either of these is like the stimulus of the lash, which keeps the poor brute working until all power is exhausted, and it falls dead; as to the brain, it means that it has worked under the stimulus of thought or alcohol, until it has no power left, has not strength left to "eat," to appropriate the nourishment which the blood contains, for its own support; as men have died for want of strength, ability to eat or swallow food, a paralysis of the muscles of deglutition, of swallowing. Hence studious men, men of great minds, often suffer from attacks of paralysis; and also, men who are engaged in vast business concerns, especially merchants, bankers, stock-brokers, many of whom in the exhaustions, bodily and mental, of business, fall into drinking habits; they drink more and more, oftener and oftener,

"TO KEEP UP,"

until the brain can bear no more, and they drop down dead of

"DISEASE OF THE HEART,"

it is charitably stated in the morning papers, but in reality of over-stimulation of the brain. This is especially true of that class of men who are never known to be actually drunk, but are "always full."

Men may also exhaust all their strength in common labor, so much of it going out through the muscles, that enough is not left in the brain to enable it to draw from the blood sufficient nutriment to sustain it, and thus the

WORKING MAN

falls a victim to paralysis.

While these things are going on in the brain, certain changes are taking place at the extreme ends of the blood-vessels of the part; they are gradually getting more and more full of blood, from their want of power — which power is also derived from the brain — to propel it, to carry it along to its proper destination; they at length become so distended that they cannot contract on their contents, and there is

CONGESTION.

In this condition the brain has power — power enough to conduct the general operation of the system — power of thought, of sight, of taste, of touch, and feeling, at least in some parts of the body; but not of touch, or feeling, or motion of the parts more particularly affected; and here comes in the philosophy of the

CURE OF PARALYSIS.

This congestion of the blood in the minute blood-vessels must be removed, which may be done in two ways. First, by medicines which will act upon the parts paralyzed directly, to stimulate them to work and pass on the blood; or which act directly on the general system, as by purgative pills, which have the effect to diminish the quantity of blood in the whole body, and proportionally in the extremities of the blood-vessels; and if that diminishes their distention to an extent which will restore to them that contractile power which carries the blood forward, the cure can be effected; by this contractile power is meant the same operation noticed when taking in the hand an India rubber bag filled with water; the contraction, the shutting of the hand, presses the water forward towards the opening.

In some cases, bleeding might accomplish the object of diminishing the amount of blood in the whole body, and, in proportion, of that in the small, congested blood-vessels; but it might at the same time diminish the vitality, the life of the system, and thus aggravate the malady, as might purgative medicines.

Other medicines might be given to stimulate the action of the parts, as alcohol, in any of its forms; but alcohol gives no strength really, adds not a particle to what was there before, but only draws in advance the strength of the next moment; when that next moment comes, its supply will have been already used, and the weakness is inevitably greater than before, may in fact be so great, that there may be no power to rise, and death follows. This is the principle of the operation of alcohol in all forms of disease; hence its use as a medicine is not philosophical, is not good practice.

Warmth and motion both tend to "move on" the congested blood; but as motion causes warmth, the actual, safe remedy is motion; and this motion implies two things in the case in hand, waste and warmth. We get warm when we exercise. We get tired by exercise. Tiredness means that a certain amount of the substance of the body has been worn from it. Motion also involves friction; that friction gives waste, as applied to the body, and also implies warmth, the evolution of heat; it is the friction involved in electricity passing through the air, that sets its oxygen on fire, and gives what we call "lightning." But in paralysis there is no power of motion in the part which most needs it; then that motion must be imparted by artificial means. In some cases the hand of one side may impart motion to ailing portions of the body; or some other person may impart that motion, as in swinging a paralyzed limb; but in order to have continuous and regular, as well as regulated motion, machinery may be devised which would admirably fulfill the requirements of a specific case. It is on this principle that Taylor's

SWEDISH MOVEMENT CURE

is founded, which is as philosophical, as, in competent hands, it is efficient, in very many apparently hopeless cases; but under this treatment it is important to keep up the general health as much as possible, by the use of

NATURAL AGENCIES

of health, such as air, exercise, food, clothing, sunshine, sleep, rest, cleanliness, and mental exhilaration, the last of which may be induced by lively company, recreation, pastimes, and intellectual employments.

If exercise enough cannot be taken to work out of the system its waste, its impurities, then it is essential that medicines should be employed which will accomplish the object; as purgative pills, to the extent of keeping up one full, free evacuation of the bowels every twenty-four hours, with the use of nutritious food, confined mainly to lean meats, coarse breads, and abundant fruits, berries, and melons. This mode of treatment, to wit, this keeping up of the general health, and at the same time, the systematic, judicious, and continuous employment of artificial movements, has, in the hands of various physicians of skill and judgment, accomplished cures almost miraculous.

#### A CASE.

A gentleman largely engaged in manufacturing and mercantile pursuits, involving great care, anxiety, and responsibility, was attacked with paralysis ; after three years of suffering, the symptoms having come on very gradually, they progressed steadily, until he had no sensation in his legs or feet; a pin thrust into the flesh up to the head was not felt. He could move his legs, but unless he kept his eye upon them and placed his feet wide apart, he could not balance himself; with the curious complication that the chafing of his pants, or the brushing of a lady's dress against them in walking, gave him great suffering. At other times he had in his legs a stinging and burning sensation, confined to little spots, as if a jet of hot steam were cast against his leg; arising, it would seem, from a congested condition of parts of the spinal column, the degree of congestion varying from time to time. By the use of certain artificial movements called vibrators, he began in about two months to have some feeling in the limbs, with less paroxysmal pains; in two months more he was able to return to business, not entirely restored, having some numbness in the feet, with occasional slight pains. After eight months he returned to his physician; his general health had been good, and he could walk more firmly. For three months more he submitted himself to the artificial movements, and returned home with all the natural movements and sensations, and with his usual strength and vigor.

Paralysis is a nervous disease, and so is

#### INSANITY.

And, as in the latter case, those who undergo treatment at once, generally recover, while if allowed to remain untreated for a year or two, or more, no treatment can avail, so it is in reference to the various forms of paralysis; those cases which are promptly treated, other things being equal, are more easily benefited, more rapidly come under the influences of the treatment, with greater promise of complete and permanent recovery. This statement is expressly made in the hope that it will attract the reader's attention to the fact, and lead him to adopt the proper means of relief and cure, without a single day's unnecessary delay.

### AN ILLUSTRATION.

A Southern gentleman, aged forty-eight, had all his life enjoyed excellent health, when, on waking up one morning, he was unable to move a single limb. He could turn his head from side to side, and that was all. He had not even the power to retain the bodily dejections. At the end of five years he could not walk straight or stand erect. In endeavoring to walk, he would take a stride twice as long as he intended, and throw his legs around, much like an old fashioned flail; his thighs were almost skin and bone; his feet could be severely pinched without feeling it. He then applied for medical advice. In a month there was no change in the symptoms; in six weeks there was great improvement; he could walk erect, there was nothing singular in his gait, and his foot regained much of its sensibility; at last reports there were reasonable hopes of his recovery. Had he applied early, there is but little doubt that he could have been cured, and remained so.

### PARALYSIS OF THE FACE, arising from a diseased condition at a particular point of the nerve of the face, is also amenable to the sàme system of treatment; that is, such voluntary and artificial motions as will aid in altering the quality and quantity of the blood in the extreme arteries and veins, so as to remove congestion and restore the parts to their healthful condition. An injury had been done to the head of a gentleman, causing a diseased condition of the nerve, or a part near it; he was confined to the house for several months, followed by paralysis of the face; there was no feeling, no power of motion in it; one whole side was distorted; the lower lid of the eye was drawn downward, while the mouth and cheek were drawn on one side. He could not close his eyelids, nor even wink, causing inflammation and constant watering of the eye, making altogether an unsightly appearance. He was entirely restored in a few months.

Slight cases of paralysis are often cured by keeping up the general health; but in all the graver cases, immediate application should be made to medical men of experience, culture, and repute, who, in a long practice and wide observation, have had large opportunities of making themselves fully acquainted with the best means of alleviation and cure.

These cases of paralysis are given to encourage the unfortunate persons who have been called to suffer such a calamity, to hope that they can be cured in three ways: —

First. In mild cases, by using all the means necessary to keeping up a high state of general health, without medicine, for a considerable time.

Second. If not materially benefited, especially if growing worse, apply promptly for medical aid.

Third. If these means do not avail in a reasonable time, within four or five months after the first attack, then resort to a system of artificial movements, not in one's own hands, but under the supervision and direction of physicians who have experience in that form of treatment.

In the progress of modern civilization, the strife for bread, for wealth, for fame, becomes more and more desperate, calling into requisition all the powers and passions of our nature, leading to superhuman efforts for success, often overpowering the energies of the system, exhausting all the vitality before a man is aware of it, to be suddenly arrested in the battle of life by the lightning stroke of paralysis. Such things are becoming more and more common, and it behooves those who are wise in time, to arrange that by forty years of age the deadly strifes of life shall cease; that fewer enterprises shall be embarked in; that less risks shall be run; that fewer debts shall be incurred; that the dues from others shall be lessened in amount and decreased in number, so as to curtail the causes of annoyance, of mental disquiet, and corroding apprehensions.

At fifty, let a man resolutely resolve that nothing shall induce him to embark in any new enterprise whatever; nor to incur a single debt more; that every week shall find him owing less and less, even if he has to live on short allowance of food, wear his coat six months longer, his hat another year. At sixty,

OWE NO MAN ANYTHING.

Allow no man to owe you a farthing, except on mortgage securities, at one third their value; and better still, have the government for a debtor, so that when the interest is due, it is certain to be paid at the day, with simply the asking for it; thus avoiding the painful necessity of urging tardy debtors, of having to press your nearest and best friend unduly; or of listening to unnumbered excuses and details of disappointments and others' woes, not your own.

It is by furling the sails of life early; by keeping things taut and snug, prepared for any storm, that man can avoid, to a large extent, that utter exhaustion and wreck of the nervous system, which entails all the horrors of a class of diseases which leaves a man only the remnant of his former self; his body a skeleton, and his mind not far from idiocy; a sorrow to kindred, a pity to strangers, and the compassion of all.

